# Diphtheria—Its Treatment

**Published:** 1863-10

**Authors:** W. A. Scott

**Affiliations:** Palmyra, Iowa


[We received the following too late for insertion in the proper section.—*Ed.*]

### DIPHTHERIA.—ITS TREATMENT.

By Dr. W. A. SCOTT.

Diphtheria Wash—℞. Golden Seal, pulv., Black Pepper, pulv., Borax, pulv., Alum, pulv., Nitrate Potassa, pulv., *aa* ʒj, Salt, ʒij. Mix. Put all into a common sized teacup and fill half full of boiling water, stir and then fill full with good vinegar. When the case is bad swab with this wash the tonsils and adjacent parts every half hour. Touch every affected spot. Let the patient swallow after each swabbing from half to two tea spoonsful of the wash. When the patient is not very bad, or is getting better, swab every hour or two, lengthening the intervals as the patient gets better.

In order to keep the diphtheria wash pure I pour the quantity I wish to use each time into a separate vessel. Wipe off any slime or matter that may adhere to the swab, each time you take it from the mouth.

℞. Sweet Oil, Spts. Turpentine, Aqua Ammonia, *aa* ʒj. Mix. Shake each time before using.

Rub this liniment on the throat, outside, once every three or four hours, and keep a flannel cloth around the neck till well. The above treatment is sufficient for all uncomplicated cases, if taken at an early stage; but if the disease has progressed, I give as a tonic, etc., to an adult, 10 drops Mur. Tr. Iron every four hours, with 2 grs. Quinine between each dose. If the disease has advanced so as to cause difficult breathing, an emetic sometimes enables the patient to throw off the membrane and get well, but such cases frequently prove fatal. For an emetic I sometimes use Syrup Ipecac, though I prefer—℞. Tinct. Lobelia, ℥iij, Tinct. Sanguinaria, ℥j. Mix. For a child one year old give a teaspoonful, in sweetened water, every ten minutes till it vomits. Keep the patient's bowels regular with Castor Oil; use no harsh cathartics; and don't be over anxious if the patient is costive. It is of great benefit to the patient to be washed off once or twice a day with tepid water, in which a little soda and salt *aa* has been put.—This has been my treatment for over two years, and I have succeeded in every case where I have been called first, (which now exceeds three hundred,) and often in other cases after the attending physician had pronounced them hopeless. I still meet with diphtheria in my daily practice, and should consider myself very unfortunate were I to lose a single case under the above treatment.

Palmyra, Iowa, Sept. 20th, 1863.

